# Improving Patch-Based Convolutional Neural Networks for MRI Brain Tumor Segmentation by Leveraging Location Information

**DOI:** 10.3389/fnins.2019.01449

**Published:** 2020-01-24

**Authors:** Po-Yu Kao, Shailja Shailja, Jiaxiang Jiang, Angela Zhang, Amil Khan, Jefferson W. Chen, B. S. Manjunath

**Affiliations:** ^1^Vision Research Lab, Department of Electrical and Computer Engineering, University of California, Santa Barbara, Santa Barbara, CA, United States; ^2^Department of Neurological Surgery, University of California, Irvine, Irvine, CA, United States

**Keywords:** gliomas, brain tumor segmentation, brain parcellation atlas, convolutional neural network, DeepMedic, 3D U-Net, ensemble learning, XGBoost

## Abstract

The manual brain tumor annotation process is time consuming and resource consuming, therefore, an automated and accurate brain tumor segmentation tool is greatly in demand. In this paper, we introduce a novel method to integrate location information with the state-of-the-art patch-based neural networks for brain tumor segmentation. This is motivated by the observation that lesions are not uniformly distributed across different brain parcellation regions and that a locality-sensitive segmentation is likely to obtain better segmentation accuracy. Toward this, we use an existing brain parcellation atlas in the Montreal Neurological Institute (MNI) space and map this atlas to the individual subject data. This mapped atlas in the subject data space is integrated with structural Magnetic Resonance (MR) imaging data, and patch-based neural networks, including 3D U-Net and DeepMedic, are trained to classify the different brain lesions. Multiple state-of-the-art neural networks are trained and integrated with XGBoost fusion in the proposed two-level ensemble method. The first level reduces the uncertainty of the same type of models with different seed initializations, and the second level leverages the advantages of different types of neural network models. The proposed location information fusion method improves the segmentation performance of state-of-the-art networks including 3D U-Net and DeepMedic. Our proposed ensemble also achieves better segmentation performance compared to the state-of-the-art networks in BraTS 2017 and rivals state-of-the-art networks in BraTS 2018. Detailed results are provided on the public multimodal brain tumor segmentation (BraTS) benchmarks.

## 1. Introduction

Glioma is a common type of brain tumor in adults originating in the glial cells that support neurons and help them function. The World Health Organization (WHO) classification system categorizes gliomas from grade I (lowest grade) through grade IV (highest grade), based upon histopathologic characteristics that predict their behavior over time (Louis et al., 2007). Low-grade gliomas (LGGs) consist of WHO-grade I tumors and WHO-grade II tumors, that tend to exhibit benign tendencies and indicate a better prognosis for the patient. WHO-grade III and IV tumors are included in high-grade gliomas (HGG) that are malignant and more aggressive. Patients with HGG had median survival time (MST) 18 months, and the MST of patients with Grade III and IV glioma were 26 and 13 months, respectively (Noiphithak and Veerasarn, 2017). Gliomas are further divided into four types of sub-regions, namely edema, non-enhancing core, necrotic core, and enhancing core based on the acuteness of the tumor cells that have different appearances in MR imaging data. However, segmenting the different sub-regions of gliomas is a daunting task because of the intrinsic heterogeneity which affects their visual appearance as well as shape. Clinically, MR images help a doctor to evaluate the tumor and plan treatment. Moreover, the treatment depends on the type, size, shape, grade, and location of the tumor, which varies widely. Consequently, this observation leads to the importance of an accurate brain tumor segmentation for better diagnosis of brain tumors. Also, the manual annotation process is time consuming and resource consuming, therefore, an automated and accurate brain tumor segmentation tool is greatly in demand.

Deep neural networks (DNNs) have achieved state-of-the-art segmentation performance on the recent Multimodal Brain Tumor Segmentation (BraTS) Challenges (Bakas et al., 2018). Kamnitsas et al. (2017a) conducted the comparative study on performance and concluded that deep learning along with ensemble learning-based methods outperform the others as they leverage the advantage of each deep learning model. Wang et al. (2017) analyzed three different binary segmentations task rather than a single multi-class segmentation task, and three different binary segmentations task has a better performance than a single multi-class segmentation task. Along this line, Isensee et al. (2017) proposed to integrate segmentation layers at different levels of optimized 3D U-Net-like architectures followed by element-wise summation. Myronenko (2018) implemented a modified decoder and encoder structure of CNN to generate dense segmentation. Likewise, Isensee et al. (2018) demonstrated that an original U-Net architecture trained with additional institution dataset improved the dice score of enhancing tumor. McKinley et al. (2018) also proposed a U-Net-like network and introduce a new loss function, a generalization of binary cross-entropy, to account for label uncertainty. Furthermore, Zhou et al. (2018) explored the ensemble of different networks including multi-scale context information, and also segmented three tumor compartments in cascade with an additional attention block.

Our recent work (Kao et al., 2018) utilizes an existing parcellation to bring location information of the brain into patch-based neural networks that improve the brain tumor segmentation performance of networks. Outputs from 26 models were averaged, including 19 different types of DeepMedics (Kamnitsas et al., 2017b) and seven different types of 3D U-Nets (Çiçek et al., 2016), to get the final tumor predictions. Different from our previous ensemble, the proposed ensemble only contains six models including three DeepMedics and three 3D U-Nets with different seed initializations that only take <1 min in the inference time. We also propose a novel two-level ensemble method which reduces the uncertainty of predictions in the first level and takes advantage of different types of models in the second level. In this paper, we also demonstrate that the proposed location fusion methods improve the segmentation performance of the single state-of-the-art patch-based network and an ensemble of multiple state-of-the-art patch-based networks. The proposed ensemble has better segmentation performance compared to state-of-the-art networks in BraTS 2017 dataset and competitive performance to the state-of-the-art networks in BraTS 2018 dataset. The main contribution of this paper is two-fold. First, it proposes a location information fusion method that improves the segmentation performance of state-of-the-art networks including DeepMedic and 3D U-Net. Second, it proposes a novel two-level ensemble method which reduces the uncertainty of prediction and leverages the advantages of different segmentation networks.

## 2. Materials and Methods

This section describes the details of (i) a proposed location information fusion method for improving brain tumor segmentation using a patch-based convolutional neural network (CNN), and (ii) a proposed ensemble learning method which takes advantage of model diversity and uncertainty reduction. This section includes the data description, data pre-processing, network architectures, training, and test procedure, proposed location information fusion method, and proposed ensemble methods. The evaluation metrics are also described at the end of this section.

### 2.1. Dataset

The Multimodal Brain Tumor Segmentation Challenges (BraTS) 2017 dataset and BraTS 2018 dataset (Menze et al., 2015; Bakas et al., 2017a,b,c) comprise clinically-acquired pre-operative multimodal MRI scans of glioblastoma (GBM/HGG) and lower-grade glioma (LGG) as training, validation and test data. There are 285 subjects in the training set and 46 and 66 subjects in the validation set of BraTS 2017 and BraTS 2018, respectively. The lesion ground-truth labels are available for the training subjects but withheld for both the validation and test subjects. MRI scans were available as native (T1), post-contrast T1-weighted (T1Gd), T2-weighted (T2), and T2 Fluid Attenuated Inversion Recovery (FLAIR) volumes. These scans were distributed after being skull-stripped, pre-processed, re-sampled, and interpolated into 1 mm isotropic resolution with an image size of 240 × 240 × 155 in *x*-, *y*-, and *z*-direction. Tumor segmentation labels were produced manually by a trained team of radiologists and radiographers. The edema was segmented primarily from T2 images, non-enhancing and enhancing the core of the tumor from T1c together with the lesions visible in T1 and necrotic core from T1c. We used the annotated and co-registered imaging datasets including the Gd-enhancing tumor, the peri-tumoral edema and the necrotic and non-enhancing tumor core for our training and test procedure.

### 2.2. Data Pre-processing

Different modalities used for mapping tumor-induced tissue changes include MR-T1, MR-T1Gd, MR-T2, and MR-FLAIR, which leads to varying intensity ranges. We first normalize each modality to a standard range of values. Each MR image is pre-processed by first clipping it at (0.2 percentile, 99.8 percentile) of non-zero voxels to remove the outliers. Subsequently, each modality is normalized individually using ${\bar{x}}_{i}=\left(x_{i}-\mu \right)/\sigma$ where *i* is the index of voxel inside the brain, ${\bar{x}}_{i}$ is the normalized voxel, *x*_*i*_ is the corresponding raw voxel, and μ and σ are the mean and standard deviation of the raw voxels inside the brain, respectively.

### 2.3. Network Architectures

Two different network architectures adapted from DeepMedic (Kamnitsas et al., 2017b) and 3D U-Net (Çiçek et al., 2016) are examined in this study. DeepMedic was initially designed for brain lesion segmentation, e.g., stroke lesions (Kamnitsas et al., 2015) and brain tumor lesions (Kamnitsas et al., 2016), and 3D U-Net which is the 3D version of U-Net (Ronneberger et al., 2015) is widely used for the volumetric image segmentation tasks (Yu et al., 2017; Li et al., 2018; Jiang et al., 2019). More details of network architectures are described below.

#### 2.3.1. Modified DeepMedic

**Figure 1:**
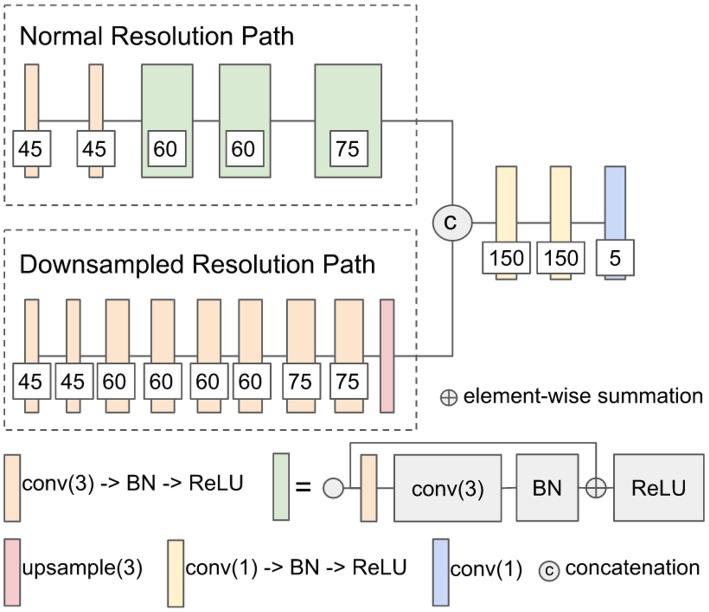
The network architecture of modified DeepMedic. conv(3), 3 × 3 × 3 convolutional layer; BN, batch normalization; upsample(3), trilinear interpolation by a factor of 3; and conv(1), 1 × 1 × 1 convolutional layer.

The first network architecture shown in Figure 1 is modified from DeepMedic (Kamnitsas et al., 2017b). The number of convolutional kernels is indicated within the white box. Batch normalization (Ioffe and Szegedy, 2015) is used. Residual connection (He et al., 2016) is used in the normal resolution path, and trilinear interpolation is used in the upsampling layer of the downsampled resolution path. The size of the receptive field of the normal resolution path is 25 × 25 × 25, and the size of the receptive field of the downsampled resolution path is 19 × 19 × 19. The receptive field of downsampled resolution path is downsampled from an image patch of size 55 × 55 × 55 by a factor of 3 in the same center as the receptive field of normal resolution path. The modified DeepMedic predicts the central 9 × 9 × 9 voxels of the receptive field of normal resolution path.

##### 2.3.1.1. Training and test procedure

The modified DeepMedic is only trained with patches that have approximately 50% foreground (lesion) and 50% background to solve the class imbalance problem, and it is trained with batch size 50. In every epoch, 20 patches are extracted from each subject. The network is trained for a total of 500 epochs. The weights of the network are updated by Adam algorithm (Kingma and Ba, 2015) with an initial learning rate of $l_{0}=10^{-3}$ following the schedule of $l_{0}\times 0.1^{\text{epoch}}$, L2 penalty weight decay of 10^−4^, and AMSGrad (Reddi et al., 2018). A standard multi-class cross-entropy loss is used. Randomly flipping in *x*-, *y*-, and *z*-axis with a probability of 50%, and random noise are applied in the data augmentation of the training procedure. At the test time, a sliding window scheme of step size 9 is used to get the tumor lesion prediction of the test subject. Training takes approximately 6 h, and a test for each subject takes approximately 24 s on an Nvidia 1080 Ti GPU and an Intel Xeon CPU E5-2696 v4 @ 2.20 GHz.

#### 2.3.2. Modified 3D U-Net

**Figure 2:**
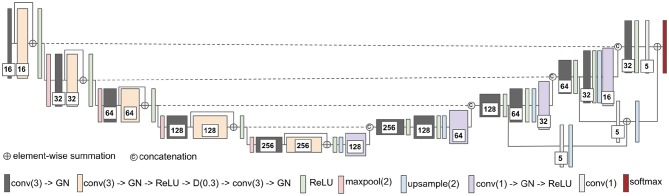
The network architecture of modified 3D U-Net. conv(3), 3 × 3 × 3 convolutional layer; GN, group normalization; D(0.3), dropout layer with 0.3 dropout rate; maxpool(2), 2 × 2 × 2 max pooling layer; and conv(1), 1 × 1 × 1 convolutional layer.

The second network architecture shown in Figure 2 is modified from 3D U-Nets (Çiçek et al., 2016). Different colors of blocks represent different types of layers. The number of convolutional kernels is indicated within the white box. Group normalization (Wu and He, 2018) is used, and the number of groups is set to 4. Residual connection (He et al., 2016) is used in the encoding path, and trilinear interpolation is used in the upsampling layer.

##### 2.3.2.1. Training and test procedure

The modified 3D U-Net is trained with randomly cropped patches of size 128 × 128 × 128 voxels and batch size 2. In every epoch, a cropped patch is randomly extracted from each subject. The network is trained for a total of 300 epochs. The weights of the network are updated by Adam algorithm (Kingma and Ba, 2015) with an initial learning rate $l_{0}=10^{-3}$ following the schedule of $l_{0}\times 0.1^{\text{epoch}}$, L2 penalty weight decay of 10^−4^, and AMSGrad (Reddi et al., 2018). For the loss function, the standard multi-class cross-entropy loss with the hard negative mining is used to solve the class imbalance problem of the dataset. We only back-propagate the negative (background) voxels with the largest losses (hard negative) and the positive (lesions) voxels to the gradients. In our implementation, the number of selected negative voxels is at most three times more than the number of positive voxels. Besides, data augmentation is not used for both training and testing. At the test time, we input the entire image of size 240 × 240 × 155 voxels into the trained 3D U-Net for each patient to get the predicted lesion mask. Training takes approximately 12.5 h, and the test takes approximately 1.5 s per subject on an Nvidia 1080 Ti GPU and an Intel Xeon CPU E5-2696 v4 @ 2.20 GHz.

### 2.4. Incorporating Location Information With Patch-Based Convolutional Neural Network

**Figure 3:**
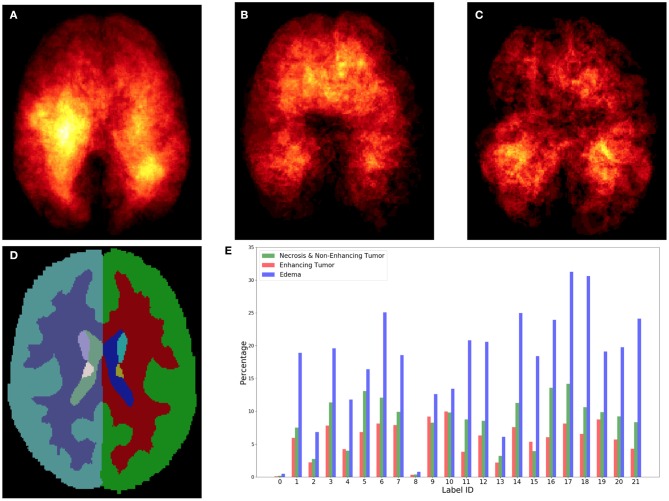
Top row shows the heatmaps of different lesion sub-regions, **(A)**: edema, **(B)**: necrosis & non-enhancing tumor, and **(C)**: enhancing tumor, from 285 training subjects of BraTS 2018 in the MNI 152 1 mm space. The brighter (yellow) voxel represents higher value. **(D)** Shows Harvard-Oxford subcortical structural atlas (Desikan et al., 2006), and **(E)** the percentage of brain lesion sub-regions observed in different parcellation regions of the Harvard-Oxford subcortical atlas from 285 training subjects of BraTS 2018. The *x*-axis indicates the brain parcellation label ID. Regions not covered by the Harvard-Oxford subcortical atlas are in label 0.

The heatmaps (see Figure 3) of different brain tumor lesion sub-regions reveal that different lesion sub-regions have different probability occurring in different locations. The heatmaps are generated by first registering the ground-truth lesions of 285 training subjects from the subject space to the MNI 152 1mm space using FMRIB's Linear Image Registration Tool (FLIRT) (Jenkinson and Smith, 2001) from FSL, extracting the binary masks of different types of lesion sub-regions from each subject, and applying element-wise summation to the same type of binary masks of each subject in the MNI 152 1mm space. However, the patch-based convolutional neural networks (CNNs), e.g., DeepMedic or 3D U-Net, do not consider location information for brain tumor segmentation. That is, the patch-based CNNs do not know location information of the input patches.

In this study, an existing brain parcellation atlas, Harvard-Oxford Subcortical atlas (see Figure 3), is used as location information of the brain for the patch-based CNN. The details of Harvard-Oxford Subcortical parcellation regions are described in Table 1. There are two main reasons for choosing this atlas: (1) this atlas covers more than 90% of a brain region, and (2) lesion information and location information are converted into this atlas (see Figure 3). The distribution in Figure 3E is calculated by dividing the total volume of the lesion sub-regions from 285 training subjects by the total volume of the corresponding brain parcellation in the MNI 152 space. Figure 3 shows that different lesion sub-regions have different probabilities happening in different parcellation regions.

**Table 1:** The label ID and corresponding brain region of Harvard-Oxford Subcortical Atlas.

**Label ID**	**Brain region**
1	Left Cerebral White Matter
2	Left Cerebral Cortex
3	Left Lateral Ventrical
4	Left Thalamus
5	Left Caudate
6	Left Putamen
7	Left Pallidum
8	Brain-Stem
9	Left Hippocampus
10	Left Amygdala
11	Left Accumbens
12	Right Cerebral White Matter
13	Right Cerebral Cortex
14	Right Lateral Ventricle
15	Right Thalamus
16	Right Caudate
17	Right Putamen
18	Right Pallidum
19	Right Hippocampus
20	Right Amygdala
21	Right Accumbens

**Figure 4:**
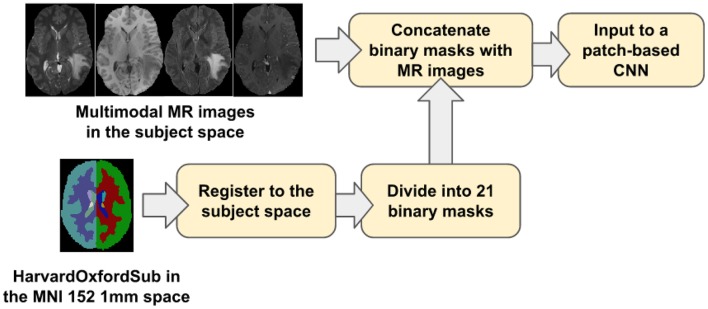
The proposed location information fusion method for brain tumor segmentation using a patch-based convolutional neural network.

Our proposed location information fusion method which is shown in Figure 4 explicitly includes location information as input into a patch-based CNN. First, the Harvard-Oxford subcortical atlas is registered to the individual subject space from MNI 152 1 mm space (Grabner et al., 2006) using FLIRT (Jenkinson and Smith, 2001) from FSL. The registered atlas is then split into 21 binary masks and concatenated with the multimodal MR images as input to a patch-based CNN for both training and test. As a result, the fused input has 25 channels. The first four channels provide the image information, and the last 21 channels contain the location information of the brain.

It is noted that the registration involving in our research only contain a linear (affline) transformation which has 9 degrees of freedom. In general, the registration should include a linear transformation followed by a deformable transformation. However, for the patient having brain lesions, a lesion mask has to be given in the deformable transformation in order to account for the effect of the lesion (Kuijf et al., 2013). The problem we have here is finding the brain tumor lesion based on the multimodal MR scan. Therefore, we are not able to use any ground truth lesion information, and the registration only contains a linear (affine) transformation.

### 2.5. Ensemble Methods

Ensemble methods aim at improving the predictive performance of a given statistical learning or model fitting technique. The general principle of ensemble methods is to construct a linear combination of some model fitting methods, instead of using a single fit of the method (Bühlmann, 2012). Ensembles have been proven to have better performance than any single model (Dietterich, 2000). Two-level ensemble approach, including the arithmetic mean and boosting, is proposed in this study, and more details of these methods are explained below.

#### 2.5.1. Arithmetic Mean

The arithmetic mean, $\bar{x}$, is the average of *n* values *x*_1_, *x*_2_, …*x*_*n*_, i.e., $\bar{x}=\left(x_{1}+x_{2}+\ldots +x_{n}\right)/n$. If we have *n* models in our ensemble, then the arithmetic mean *P* is defined by the formula:

(1) $$\begin{array}{l} P=\frac{1}{n}\overset{n}{\underset{i=1}{\sum }}p_{i}=\frac{p_{1}+p_{2}+\ldots +p_{n}}{n} \end{array}$$

where *p*_*i*_ is the probability map of model *i*. The arithmetic mean ensemble method reduces the uncertainties of different models.

#### 2.5.2. XGBoost

Boosting algorithms are widely used in machine learning to achieve state-of-art performance. It improves the prediction of the models by training the base learners sequentially to improve their predecessor. There are different boosting algorithms such as AdaBoost (Freund and Schapire, 1997; Hastie et al., 2009), short for Adaptive Boosting, and Gradient Boosting (Friedman, 2001, 2002). AdaBoost tunes the weights for every incorrect classified observation at every iteration while Gradient Boosting tries to fit the new predictor to the residual errors made by the previous predictor. Both of the boosting algorithms are generally very slow in implementation and not very scalable. Chen and Guestrin (2016) described a scalable tree boosting system called XGBoost which is an implementation of gradient boosted decision trees that are efficient in run-time and space complexity. It also supports parallelization of tree construction, distributed computing for training very large models, out-of-core computing for very large datasets that do not fit into memory and cache optimization to make the best use of hardware. These features make XGBoost ideal for our purpose of study in brain tumor segmentation, therefore, it is used in our study.

#### 2.5.3. Two-Level Ensemble Approach: Arithmetic Mean and XGBoost

**Figure 5:**
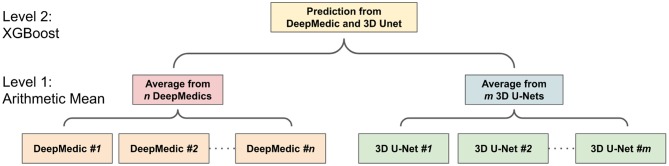
The workflow of two-level ensemble approach.

The ensemble of multiple identical network architectures with different seed initializations has been proven to reduce the uncertainty of models and improve the segmentation performance (Lakshminarayanan et al., 2017). Moreover, Dietterich (2000) demonstrated that the boosting algorithm has the best performance compared to bagging and randomized trees. Inspired by their works, we propose a two-level ensemble approach shown in Figure 5 that averages the probability maps from the same type of models in the first level and then boosts the averaged probability maps from different models by using the XGBoost algorithm in the second level. We have examined three different classification strategies in the second level, and these classification strategies are based on multi-class classification and binary class classification. More details are described in sections 2.5.3.1 and 2.5.3.2.

##### 2.5.3.1. Multi-class classification

The multi-class classification problem refers to classifying voxels into one of the four classes. It produces segmentation labels of the background and different glioma sub-regions that include: (1) the enhancing tumor, (2) the edema, and (3) the necrosis & non-enhancing tumor. Since XGBoost is known to produce better results in different machine learning problems (Nielsen, 2016), XGBoost is used in our multi-class classification problem with the softmax function objective. The softmax function σ is defined by

$$\begin{array}{l} \sigma {\left(\text{z}\right)}_{i}=\frac{e^{z_{i}}}{\sum_{j=1}^{k}e^{z_{j}}}\text{ for }i=1,\ldots ,K\text{ and }\\ \;\text{z}=\left(z_{1},\ldots ,z_{K}\right)\in {\mathbb{R}}^{K}. \end{array}$$

where *K* is the number of classes in the classification problem. Using the softmax objective function, we get a neural network that models the probability of a class *z*_*i*_ as multinominal distribution.

##### 2.5.3.2. Binary classifications

The multi-class classification problem can be reduced to several binary classification problems where each binary classifier is trained to classify voxels into two classes. There are two different approaches, one-versus-all and one-versus-one, to perform such a transformation. For a *k*-class problem, the one-versus-all method trains *k* different binary classifiers where the two-class classifier *C*_*i*_ learns to distinguish the class *i* from all the other *k* − *i* classes.

$$\begin{array}{l} C_{+}=C_{i}\text{ and }C_{-}=\left\{C_{j}|j=1,\cdots ,K,j\neq i\right\} \end{array}$$

One-vs.-one approach is based on training *k*×(*k* − 1)/2 classifiers, where each classifier learns to distinguish 2 classes only.

$$\begin{array}{l} C_{+}=C_{i}\text{ and }C_{-}=\left\{C_{j}|j\neq i\right\} \end{array}$$

where *C*_+_ and *C*_−_ are the two classes of the binary class classification problem.

### 2.6. Evaluation Metrics

Two evaluation metrics, dice similarity score (DSC) and Hausdorff distance, are commonly used in the brain tumor segmentation problem. DSC is used to measure the similarity of the predicted lesions and ground-truth lesions, and Hausdorff distance is used to measure how far the predicted lesions are from the ground-truth lesions. More details of these two evaluation metrics are explained in the following sections.

#### 2.6.1. Dice Similarity Score

Dice similarity score (DSC) is a statistic used to measure the similarity of two sets. It is defined as

(2) $$\begin{array}{l} DSC=\frac{2|G\cap P|}{\left|G|+|P\right|} \end{array}$$

where |*G*| and |*P*| are the number of voxels in the ground-truth and prediction, respectively. DSC ranges between 0 and 1 (1 means perfect matching).

#### 2.6.2. Hausdorff Distance

Hausdorff distance *d_H_*(*X, Y*) measures how far two subsets {*X, Y*} of a metric space are from each other. It is defined as

(3) $$\begin{array}{l} d_{H}\left(X,Y\right)=\max\left\{\underset{x\in X}{\sup}\underset{y\in Y}{\inf}d\left(x,y\right),\underset{y\in Y}{\sup}\underset{x\in X}{\inf}d\left(x,y\right)\right\} \end{array}$$

where *d* is the Euclidean distance, sup is the supremum, and inf is the infimum. Hausdorff distance ranges from 0 to infinity (0 means perfect matching). In this study, 95 percentile of Hausdorff distance (HD95) is used to disregard the outliers.

## 3. Experiments and Results

In this section, we demonstrate the advantage of the proposed location information fusion method and the proposed two-level ensemble learning method. In Experiment 1, we first examine the segmentation performance of the proposed location information fusion method on a single model. In Experiment 2, we examine the performance of the proposed location information fusion method on an ensemble of the same type of models. In Experiment 3, we examine different ensemble methods that predict the final brain tumor lesions based on the output probability maps from DeepMedics and 3D U-Nets. In Experiment 4, we compare the segmentation performance of the proposed method with state-of-the-art methods. The details of each experiment and experimental results are described in the following sections.

### 3.1. Experiment 1: Location Information Fusion Method on a Single Model

In the first experiment, we would like to examine the performance of the proposed location information fusion method on a single patch-based neural network. We first train a DeepMedic and a 3D U-Net using only multimodal MR images. Thereafter, we train another identical DeepMedic and another identical 3D U-Net with multimodal MR images and binary brain parcellation masks. BraTS 2018 training set is used to train the models with five-fold cross-validation, and the BraTS 2018 validation set is used as the test set. The experimental results are shown in Table 2.

**Table 2:** Results of the first experiment on the BraTS 2018 validation set.

**Model description**	**DSC_ET**	**DSC_WT**	**DSC_TC**	**HD95_ET**	**HD95_WT**	**HD95_TC**
DeepMedic	78.1(25.4)	89.5(6.8)	81.4(21.3)	4.21(8.19)	10.60(15.30)	9.90(20.13)
DeepMedic + BP	**79.0(22.6)**	**89.6(6.4)**	81.3(21.8)	**3.78(7.23)**	**8.87(15.23)**	**6.55(6.81)**
3D U-Net	74.9(25.8)	89.7(7.7)	76.6(20.3)	5.85(9.50)	4.88(4.41)	10.46(13.51)
3D U-Net + BP	**76.4(25.4)**	**90.1(6.4)**	**76.9(24.4)**	**5.48(9.50)**	**4.87(6.28)**	**10.07(13.99)**

The results are reported as mean (standard deviation). Bold numbers highlight the improved results with additional brain parcellation masks within the same type of model.

### 3.2. Experiment 2: Location Information Fusion Method on an Ensemble

In the second experiment, we would like to examine the performance of the proposed location information fusion on the ensemble of DeepMedics and the ensemble of 3D U-Nets. Each ensemble has identical network architectures with different seed initializations, and the output of the ensemble is the arithmetic mean from networks. We first train ensembles of DeepMedics without additional brain parcellation masks. Thereafter, we train ensembles of 3D U-Nets without additional brain parcellation masks. In the end, we train another identical ensemble of DeepMedics and another identical ensemble of 3D U-Nets with additional brain parcellation masks. BraTS 2018 training set is used to train the models with five-fold cross-validation, and the BraTS 2018 validation set is used as the test set. The experimental results are shown in Table 3.

**Table 3:** Results of the second experiment on the BraTS 2018 validation set.

**Ensemble description**	**DSC_ET**	**DSC_WT**	**DSC_TC**	**HD95_ET**	**HD95_WT**	**HD95_TC**
DeepMedic	79.7(23.6)	90.0(6.8)	81.4(22.1)	3.94(7.77)	7.44(13.36)	8.88(14.03)
DeepMedic + BP	78.4(25.3)	**90.2(6.4)**	**81.8(21.9)**	**3.37(5.18)**	**5.64(7.53)**	**7.01(12.29)**
3D U-Net	77.6(24.2)	90.0(9.0)	78.0(21.2)	5.01(9.22)	4.39(4.05)	9.77(13.60)
3D U-Net + BP	77.4(25.1)	**90.4(6.6)**	**79.3(22.4)**	**4.25(8.31)**	4.59(6.29)	**9.66(14.20)**

The results are reported as mean (standard deviation). Bold numbers highlight the improved results with additional brain parcellation masks within the same type of ensemble.

### 3.3. Experiment 3: Different Ensemble Methods

In the third experiment, we would like to exam the performance of different ensemble methods including arithmetic mean and two-level ensemble approaches described in section 2.5. We first train three identical DeepMedics with additional brain parcellation channels and different seed initializations. We also train three identical 3D U-Nets with additional brain parcellation channels and different seed initializations. Then, we apply different ensemble methods on the probability maps from these models to generate the final tumor segmentation mask. More details of different ensemble methods are described below.

#### 3.3.1. Experiment 3.1: Arithmetic Mean

In this experiment, the final tumor segmentation mask is directly generated by averaging the probability maps from three DeepMedics and three 3D U-Nets. BraTS 2018 training set is used to train the models with five-fold cross-validation, and the BraTS 2018 validation set is used as the test set. The experimental results are shown in Table 4.

**Table 4:** Results of the third experiment on BraTS 2018 validation set.

**Ensemble methods**	**DSC_ET**	**DSC_WT**	**DSC_TC**	**HD95_ET**	**HD95_WT**	**HD95_TC**
Arith. mean	**78.3(25.4)**	90.6(6.4)	81.3(21.8)	3.72(7.90)	**4.35(6.21)**	7.77(13.45)
TLMC	78.3(25.5)	90.7(6.3)	81.0(22.1)	**2.81(3.55)**	4.38(6.26)	7.80(13.49)
TLBC	76.6(26.8)	90.7(6.2)	82.2(21.2)	7.93(2.66)	4.39(6.27)	8.34(16.98)
TLFC	78.2(25.6)	**90.8(6.1)**	**82.3(21.2)**	2.96(3.80)	4.39(6.22)	**6.91(12.64)**

The results are reported as mean (standard deviation). Bold numbers highlight the best performance between different ensemble methods.

#### 3.3.2. Experiment 3.2: Two-Level Ensemble: Multi-Class Classification

In this experiment, we directly apply an XGBoost classifier on the probability maps from three DeepMedics and three 3D U-Nets. The input vector of the XGBoost classifier has 10 dimensions (5-class probability maps from 2 ensembles of the same type of models). The XGBoost classifier outputs the 5-class labels which contain a background (label 0), enhancing tumor (label 1), edema (label 2), and necrosis & non-enhancing tumor (label 4). BraTS 2018 training set is used to train the models with five-fold cross-validation, and the BraTS 2018 validation set is used as the test set. The experimental results are shown in Table 4 as TLMC.

#### 3.3.3. Experiment 3.3: Two-Level Ensemble: Binary Classification

**Figure 6:**
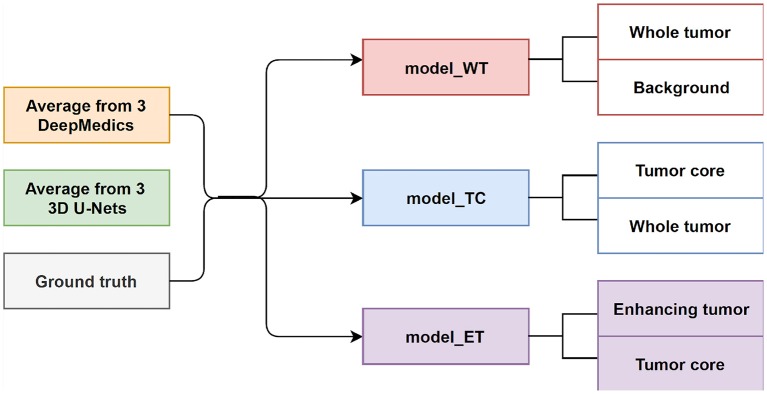
The training workflow of two-level binary classification approach.

In this experiment, we train three XGBoost binary classifiers on the resulting probability maps generated from three DeepMedics and three 3D U-Nets in the first level. During training, each classifier uses a one-vs.-one approach to distinguish between two binary classes. We trained three different models namely, model_WT (whole tumor), model_TC (tumor core), and model_ET (enhancing tumor) as shown in Figure 6.

$$\begin{array}{l} \text{For model\_WT: }C_{+}=C_{WT}\text{ and }C_{-}=C_{background}\\ \text{For model\_TC: }C_{+}=C_{TC}\text{ and }C_{-}=C_{WT}\\ \text{For model\_ET: }C_{+}=C_{ET}\text{ and }C_{-}=C_{TC} \end{array}$$

The whole tumor region is the union of edema, non-enhancing tumor & necrosis, and enhancing tumor, and the tumor core regions is the union of edema and non-enhancing tumor & necrosis. Therefore, the tumor core class is a subset class of the whole tumor, and the enhancing tumor class is a subset of the tumor core class. For prediction, we feed the average probability maps from three DeepMedics and three 3D U-Nets to the three models. The input vector has 10 dimensions (5-class probability maps from 2 ensembles of the same type of models). The model_WT classifies the voxels into the whole tumor and background. For model_TC, we feed the probability maps of such voxels that are classified as the whole tumor from the experiment in section 3.3.1. For model_ET, we feed the probability maps of such voxels that are classified as tumor core from the previous prediction in the experiment. BraTS 2018 training set is used to train the models with five-fold cross-validation, and the BraTS 2018 validation set is used as the test set. The experimental results are shown in Table 4 as TLBC.

#### 3.3.4. Experiment 3.4: Two-Level Ensemble: Fusion Classifications

**Figure 7:**
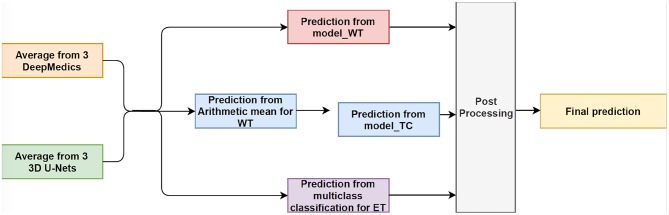
The workflow of Fusion Classification method. For post processing step, we classify the voxels into three classes with background, whole tumor, tumor core, and necrosis & non-enhancing tumor in decreasing order of priority. For example, if a voxel is classified into both whole tumor and tumor core, we give the final label as that of tumor core according to the preference mentioned before.

This is the final experiment to integrate the methods from the previous experiments. We observe that while the experiment in section 3.3.3 performs best for classifying voxels into the background, whole tumor and tumor core, the experiment in section 3.3.1 has the best performance on necrosis & non-enhancing tumor. We use model_WT, model_TC, and multi-class classifier model for the fusion model. For prediction, we feed the average probability maps from three DeepMedics and three 3D U-Nets to the three models. The input vector has 10 dimensions (5-class probability maps from 2 ensembles of the same type of models). The model_WT classifies the voxels into the whole tumor and background. For model_TC that is trained to classify voxels into the whole tumor and tumor core, we feed the probability maps of such voxels that are classified as the whole tumor from the experiment in section 3.3.1. For necrosis & non-enhancing tumor class, we feed the probability maps to the multi-class classifier as in section 3.3.2. To merge the three different predicted results, we classify the voxels into three classes with background, whole tumor, tumor core, and necrosis & non-enhancing tumor in decreasing order of priority. For example, if a voxel is classified into both whole tumor and tumor core, we give the final label as that of tumor core according to the preference mentioned before. Therefore integrating these two gives the effective scores as shown in Table 4 as TLFC. BraTS 2018 training set is used to train the models with five-fold cross-validation, and the BraTS 2018 validation set is used as the test set. The workflow of fusion classification is shown in Figure 7.

### 3.4. Experiment 4: Compare to the State-of-the-Art Methods

In this experiment, we compare the brain tumor segmentation performance of the proposed method described in section 3.3.4 with the state-of-the-art methods on both BraTS 2017 and BraTS 2018 dataset. The quantitative results are shown in Table 5.

**Table 5:** The first three rows show the results of our proposed method and the state-of-the-art methods on the BraTS 2017 validation set, and the bottom four rows show the results of our proposed method and the state-of-the-art methods on BraTS 2018 validation set.

		**DSC**			**HD95**		
**Methods**	**No. of models**	**ET**	**WT**	**TC**	**ET**	**WT**	**TC**
Kamnitsas et al. (2017a)	7	73.8	90.1	**79.7**	4.50	**4.23**	**6.56**
Isensee et al. (2017)	5	73.2	89.6	**79.7**	4.55	6.97	9.48
Proposed method	6	**74.3**	**90.4**	78.5	**3.49**	4.46	8.45
Myronenko (2018)	10	**82.3**	**91.0**	**86.6**	3.93	4.52	**6.85**
Isensee et al. (2018)	10	81.0	90.8	85.4	**2.54**	4.97	7.04
Kao et al. (2018)	26	78.8	90.5	81.3	3.81	**4.32**	7.56
Proposed method	6	78.2	90.8	82.3	2.96	4.39	6.91

*The results are reported as mean. Bold numbers highlight the best performance in each dataset. These results are directly copied from their paper*.

## 4. Discussion and Conclusion

Due to the computational limitation of training the state-of-the-art networks using GPU, we are not able to input the whole brain volume of size 240 × 240 × 155 to a neural network for training purposes. Alternatively, we randomly crop sub-regions of the brain and input these sub-regions to the neural network for training. For the current patch-based neural networks, we noted that these neural networks lack location information of the brain for both training and test procedure. That is, these patch-based neural networks do not have the information about where the patch comes from the brain. Therefore, we proposed the location fusion method which explicitly carries location information of the brain into patch-based neural networks such as 3D U-Net and DeepMedic. An existing structural brain parcellation atlas, HarvardOxford Sub-cortical Atlas, is used as additional location information to these patch-based neural networks in both training and test.

**Figure 8:**
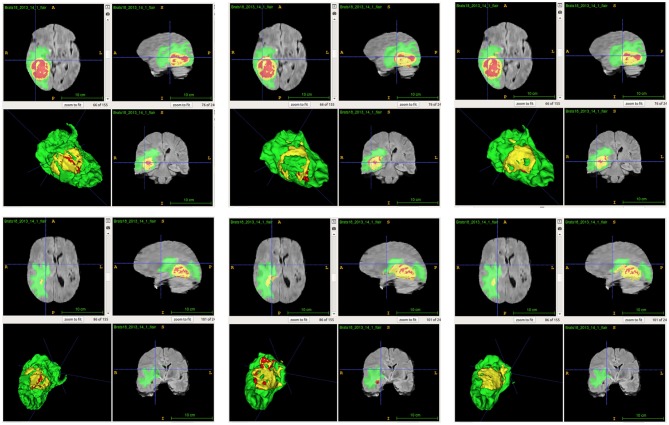
Examples of predictions from single model with different inputs. Top row shows the predictions from DeepMedic, and bottom row shows the predictions from 3D U-Net (from left to right: ground-truth lesions, prediction from single model, and prediction from single model with additional brain parcellation masks.) Red: enhancing tumor, yellow: necrosis & non-enhancing tumor, and green: edema. ITK-SNAP (Fedorov et al., 2012) is used to visualize the MR images and lesion masks.

**Figure 9:**
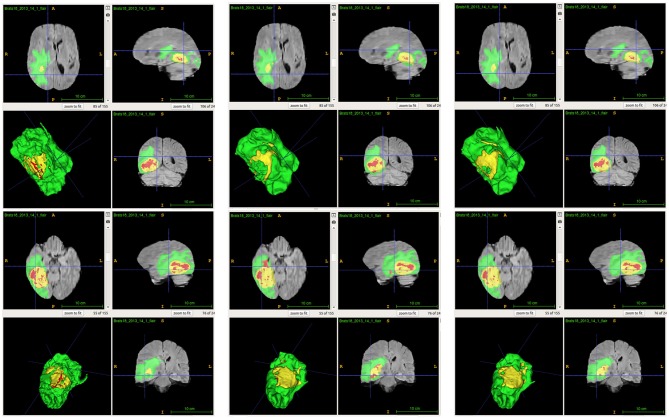
Examples of predictions from ensemble with different inputs. Top row shows the predictions from ensemble of DeepMedics, and bottom row shows the predictions from ensemble of 3D U-Nets (from left to right: ground-truth lesions, prediction from ensemble, and prediction from ensemble with additional brain parcellation masks.) Red: enhancing tumor, yellow: necrosis & non-enhancing tumor, and green: edema. ITK-SNAP (Fedorov et al., 2012) is used to visualize the MR images and lesion masks.

From Table 2, we demonstrate that the proposed location fusion method improves the brain tumor segmentation performance of both a single state-of-the-art model. We also demonstrate that the proposed location fusion method improves the ensemble of multiple same types of state-of-the-art models in Table 3. The proposed location fusion method yields a smoother prediction for both 3D U-Net and DeepMedic compared to the resulting prediction without location information (see Figures 8, 9).

**Figure 10:**
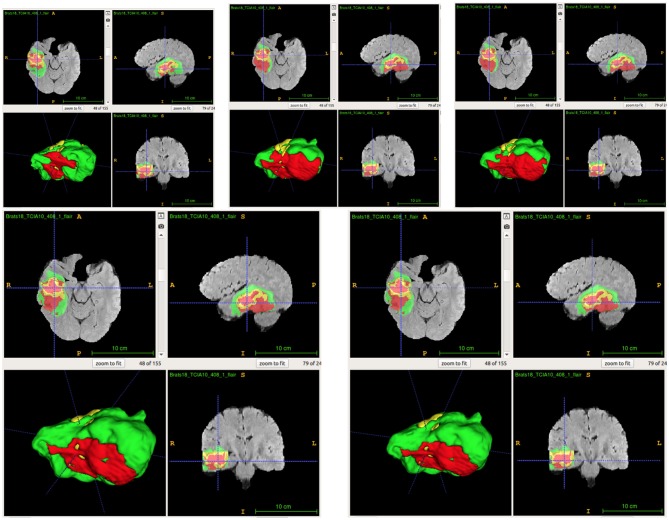
Examples of predictions from different ensemble methods. The top left image shows the ground-truth lesion mask, and the top middle image shows the predictions using the arithmetic mean. The top right image shows the prediction using a two-level multi-class classification (TLMC) method. The bottom left image shows the prediction using a two-level binary classification (TLBC) method, and the bottom right image shows the prediction using a two-level fusion classification (TLFC) method. Red: enhancing tumor, yellow: necrosis & non-enhancing tumor, and green: edema. ITK-SNAP (Fedorov et al., 2012) is used to visualize the MR images and lesion masks.

From Table 4, the proposed ensemble method, two-level fusion classification (TLFC) method, has the best performance compared to other ensemble methods including arithmetic mean, two-level multi-class classification (TLMC), and two-level binary classification (TLBC). TLFC takes advantage of TLMC and TLBC. Moreover, Figure 10 shows the predictions of brain tumor lesions from different ensemble methods, and TLFC method has the best performance among other methods.

From Table 5, the proposed method has the best tumor segmentation performance compared to other state-of-the-art methods in BraTS 2017 with a similar number of models in the ensemble. Also, the proposed method has a competitive tumor segmentation performance compared to other state-of-the-art methods in BraTS 2018 with fewer models in the ensemble. It is noted that the model of Myronenko (2018) requires a large amount of GPU memory (32 GB) for training, and Isensee et al. (2018) trained the models with additional public and institutional data. In addition, Myronenko (2018) and Isensee et al. (2018) have 10 models in their ensemble but our proposed ensemble only has six models. The proposed ensemble has much fewer models with a better segmentation performance compared to our previous work which has 26 models (Kao et al., 2018). The test time of our previous ensemble takes approximately 30 min on an Nvidia 1080 Ti GPU and an Intel Xeon CPU E5-2696 v4 @ 2.20 GHz. However, the proposed ensemble only takes approximate 3 min on the same infrastructure. Our previous ensemble ranked 6th out of 63 teams in BraTS 2018 segmentation challenge, and the proposed ensemble even has a better performance and less inference time compared to the previous ensemble.

Summarizing, in this paper we proposed a novel method to integrate location information about the brain into a patch-based neural network for improving brain tumor segmentation. Our experimental results demonstrate that the proposed location information fusion approach improves the segmentation performance of the baseline models including DeepMedic and 3D U-Net. Moreover, the proposed location information fusion method can be easily integrated with other patch-based network architectures to potentially enhance their brain tumor segmentation performance. We also proposed a two-level fusion classification method which reduces the uncertainty of prediction in the first level and takes advantage of different types of models in the second level. Also, the proposed ensemble method can also be easily integrated with more different types of neural networks. The proposed ensemble helps the neurologists on delineating brain tumors and improves the quality of the neuro-surgery.

## Data Availability Statement

The dataset analyzed for this study can be found in the BraTS 2017 and BraTS 2018 of CBICA Image Processing Portal [https://ipp.cbica.upenn.edu/].

## Author's Note

All data are made available online with a permissive copyright license (CC-BY-SA 4.0), allowing for data to be shared, distributed, and improved upon.

## Author Contributions

P-YK, SS, and JJ designed the study and algorithm with BM guiding the research and analyzed and interpreted the data. P-YK collected the data. P-YK and SS sourced the literature and wrote the draft. P-YK, SS, JJ, AZ, AK, JC, and BM edited the manuscript. JC provided medical and clinical insights.

### Conflict of Interest

The authors declare that the research was conducted in the absence of any commercial or financial relationships that could be construed as a potential conflict of interest.
