# Corrigendum: Improving Patch-Based Convolutional Neural Networks for MRI Brain Tumor Segmentation by Leveraging Location Information

**DOI:** 10.3389/fnins.2020.00328

**Published:** 2020-04-15

**Authors:** Po-Yu Kao, Shailja Shailja, Jiaxiang Jiang, Angela Zhang, Amil Khan, Jefferson W. Chen, B. S. Manjunath

**Affiliations:** ^1^Vision Research Lab, Department of Electrical and Computer Engineering, University of California, Santa Barbara, Santa Barbara, CA, United States; ^2^Department of Neurological Surgery, University of California, Irvine, Irvine, CA, United States

**Keywords:** gliomas, brain tumor segmentation, brain parcellation atlas, convolutional neural network, DeepMedic, 3D U-Net, ensemble learning, XGBoost

An author's name was incorrectly published as “**Fnu Shailja**.” It should be “**Shailja Shailja**.”

The authors apologize for this error and state that this does not change the scientific conclusions of the article in any way. The original article has been updated.

